# Did glacial advances during the Pleistocene influence differently the demographic histories of benthic and pelagic Antarctic shelf fishes? – Inferences from intraspecific mitochondrial and nuclear DNA sequence diversity

**DOI:** 10.1186/1471-2148-7-220

**Published:** 2007-11-12

**Authors:** Karel Janko, Guillaume Lecointre, Arthur DeVries, Arnaud Couloux, Corinne Cruaud, Craig Marshall

**Affiliations:** 1 Laboratory of Fish Genetics, Institute of Animal Physiology and Genetics, Academy of Sciences of the Czech Republic, Rumburská 89, 27721 Libechov, Czech Republic; 2 UMR CNRS 7138 "Systématique, Adaptation, Evolution", Département "Systématique et Evolution", Muséum National d'Histoire Naturelle, 43 rue Cuvier 75231 Paris cedex 05, France; 3 Animal Biology, University of Illinois at Urbana-Champaign, 524 BH, 407 S. Goodwin, Urbana, Il 61801, USA; 4 Genoscope. Centre National de Sequençage. 2, rue Gaston Crémieux, CP5706, 91057 Evry Cedex, France; 5 Department of Biochemistry, University of Otago, P.O. Box 56, Dunedin, New Zealand

## Abstract

**Background:**

Circum-Antarctic waters harbour a rare example of a marine species flock – the Notothenioid fish, most species of which are restricted to the continental shelf. It remains an open question as to how they survived Pleistocene climatic fluctuations characterised by repeated advances of continental glaciers as far as the shelf break that probably resulted in a loss of habitat for benthic organisms. Pelagic ecosystems, on the other hand, might have flourished during glacial maxima due to the northward expansion of Antarctic polar waters. In order to better understand the role of ecological traits in Quaternary climatic fluctuations, we performed demographic analyses of populations of four fish species from the tribe Trematominae, including both fully benthic and pelagic species using the mitochondrial cytochrome b gene and an intron from the nuclear S7 gene.

**Results:**

Nuclear and cytoplasmic markers showed differences in the rate and time of population expansions as well as the likely population structure. Neutrality tests suggest that such discordance comes from different coalescence dynamics of each marker, rather than from selective pressure. Demographic analyses based on intraspecific DNA diversity suggest a recent population expansion in both benthic species, dated by the cyt b locus to the last glacial cycle, whereas the population structure of pelagic feeders either did not deviate from a constant-size model or indicated that the onset of the major population expansion of these species by far predated those of the benthic species. Similar patterns were apparent even when comparing previously published data on other Southern Ocean organisms, but we observed considerable heterogeneity within both groups with regard to the onset of major demographic events and rates.

**Conclusion:**

Our data suggest benthic and pelagic species reacted differently to the Pleistocene ice-sheet expansions that probably significantly reduced the suitable habitat for benthic species. However, the asynchronous timing of major demographic events observed in different species within both "ecological guilds", imply that the species examined here may have different population and evolutionary histories, and that more species should be analysed in order to more precisely assess the role of life history in the response of organisms to climatic changes.

## Background

The Antarctic and its surrounding ocean are mostly considered as a system isolated from the rest of world by the Antarctic Polar Front (APF), a circum-Antarctic current extending to about 1000 m depth, which greatly reduces the exchange of surface waters and marine organisms [1]. Despite some interconnections of marine biota from north and south of the APF [2-4], it seems likely that evolution in isolation within the APF resulted in cladogenetic events that lead to the formation of endemic taxa [5-9]. Organisms within the APF had to contend with freezing water temperatures and the potential eradication of their habitat by ice sheet advance and grounding, a phenomenon that that affects the Antarctic ecosystem on a scale much greater than elsewhere.

Antarctic ice sheet extension over the continental shelf during the glacial maxima [10] and the related intense flows of debris [11], extensively reduced the habitat suitable for benthic biota. In consequence, these species were either fragmented into geographically restricted glacial refuges, or escaped to a greater depths [12]. In contrast, pelagic ecosystems are likely to be less affected and might have even experienced a population expansion during glacial maxima as suggested e.g. by the Northward shift of the APF during last glacial maximum (LGM) [[13]; although this scenario is still debated] or by Northward expansions of Antarctic plankton [14] and increased ecosystem productivity [15,16]. Such a glacial-mediated eradication hypothesis provides us with testable predictions about the evolution of benthic and pelagic biota.

A coalescent population genetic model constitutes a framework for the development of efficient tests (either analytical or based on computer simulations), from which we may draw inferences about the forces affecting the evolution of populations including selection, population structuring, and demographic fluctuations. Many methods using different assumptions and types of sequence information have been proposed to detect the traces of population growth from DNA sequence data (for review see [17]). Recent advances in computational methods have also given rise to demographic inference tools that take into account phylogenetic relationships among haplotypes (see e.g. [18]). It is also possible to test for scenarios more complicated than simple population growth, such as a bottleneck, characterised by drop in population size at some time with a reduction of diversity, followed by a population expansion [19].

Only a few studies of the demographic histories of Antarctic marine and coastal organisms have been done because of the difficulties in obtaining samples. Studies using historical demographic inference tools have so far found that population expansions are linked to deglaciations for coastal populations of Collembolans and penguins [20,21] and indicated population expansions of pelagic krill [22] and fish [16], putatively correlated with the onset of ice sheet expansion.

Notothenioid fish occupy an interesting position in evolutionary research because of an adaptive radiation thought to be associated with the onset of freezing water temperatures during the Miocene [12]. It seems plausible that the radiation had a high-Antarctic origin [rev. in [23]] probably associated with niche opening after the loss of a tertiary fish fauna unable to cope with low temperatures in the Miocene. It is proposed that a putative notothenioid benthic ancestor that possessed antifreeze glycoproteins (AFGP; [24]) protecting against the growth of ice crystals in the blood or gut was able to uniquely exploit now vacant ecological niches. This led to a radiation of many species strikingly diverse in morphology and life-history, ranging from small bottom feeders to neutrally-buoyant pelagic species more than two metres in length. There remains doubt as to about the timing of the radiation of AFGP-bearing Notothenioidei, which is variously estimated to have occurred from 11 millions of years ago (Mya) [25] to 21 Mya [5], or 24 Mya [26]. Furthermore, at least three recent radiations appeared within the Notothenioidei, giving rise to the Channichthyid-Bathydraconid subfamily crown group [27,28] and to the tribe Trematominae [29,30].

Despite much work into the phylogenetic relations of notothenioids and their physiological adaptations and ecology [e.g. [23,31,32]], little is known of the microevolutionary patterns of these fishes. To investigate the role of glacial cycles and the subsequent habitat changes, we studied the genetic variability and historical demography of four notothenioid species belonging to the tribe Trematominae; *Trematomus bernacchi, T. pennelli, Pagothenia borchgrevinki *and *T. newnesi*. This tribe is thought to result from an adaptive radiation roughly correlated with Pliocene warming about 3.5 My ago [29], or dated at 9.8 My ago [[26]: Table 1 line L]. All four species are distributed in the high Antarctic zone, but with different life-histories. *Trematomus bernacchii *and *T. pennelli *are bottom feeding stationary fish with demersal eggs and some kind of parental care and pelagic larvae, whereas *P. borchgrevinki *and *T. newnesi *are cryopelagic (i.e. living in platelet ice) and semipelagic, respectively, with pelagic larvae [33,34]. We were particularly interested in the responses of these four species to well-documented climatic changes that are likely to have affected all the Southern Ocean fauna. We predict that benthic fish species are more prone to local extinctions caused by glacial advance onto the shelf, than are pelagic or semipelagic fish, and therefore benthic species are likely to have passed through more severe and recent population bottlenecks during glacial maxima than have pelagic or semipelagic species.

**Table 1:** Geographical origin of the samples of used in this study

	CR	CA	TNB	CH	TA	SSh	Total
*Trematomus bernacchi*	11	11	11	5	11	12	61
*Trematomus pennelli*	11	6	-	8	19	0	44
*Pagothenia borchgrevinki*	-	33	-	-	5	0	3
*Trematomus newnesi*	-	9	-	8	17	15	36

(CR = Cape Roberts, CA = Cape Armitage, TNB = Terra Nova Bay, CH = Cape Hallett, TA = Terre Adélie, SH = South Shetlands).

## Methods

**Figure 1:**
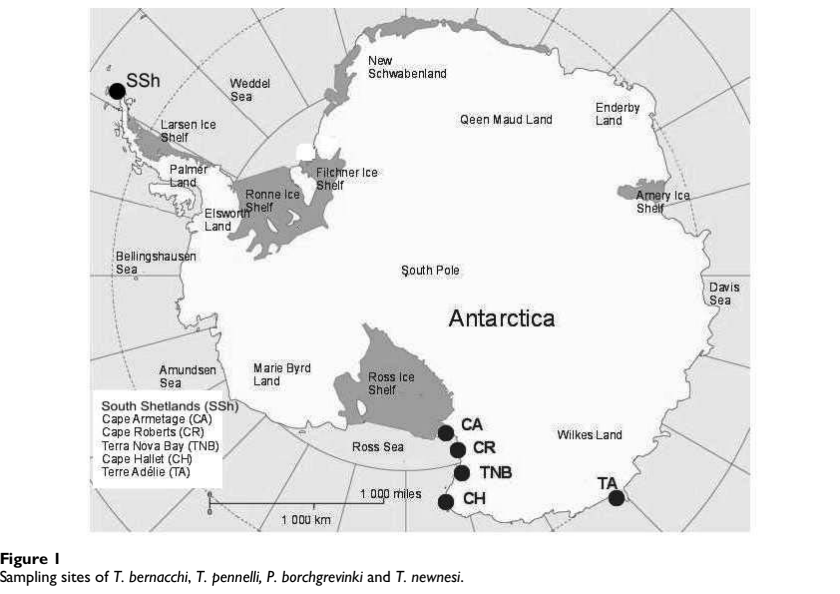
Sampling sites of *T. bernacchi*, *T. pennelli, P. borchgrevinki *and *T. newnesi*.

Collected samples are summarized in the Figure 1 and Table 1.

### DNA extraction, amplification and sequencing analyses

Total genomic DNA was extracted from muscle or fin tissues using the DNeasy tissue kit (Qiagen) following the manufacturer's specifications. A 600 bp fragment of cytochrome *b *(cyt *b*) was amplified in both species by polymerase chain reaction (PCR) using primers CytB-U15786-Tremato (5'- TGAGGkGGrTTTTCGGTAGATA-3') and CytB-L16317-Tremato (5'- GATrTAnGGrTCCTCAaCGGG-3'). Polymerase chain reactions were performed according to the published protocol [28]. The first intron in the S7 gene was amplified under following conditions: primers S7RPEX1F (TGGCCTCTTCCTTGGCCGTC) and S7RPEX2R (AACTCGTCTGGCTTTTCGCC) [35]; the reaction volume of 25 μl contained 2,5 μl of reaction buffer, 1.5 mM MgCl_2_, 1 μl of A, T, C, G nucleotide mix (0.01 M), 0.5 μl of each primer (0.01 M), 1.25 units of Taq polymerase (TopBio) and ~100 ng of genomic DNA; thermal cycling consisted of one initial cycle of denaturation at 94°C for 2 min, followed by 34 cycles of denaturation at 94°C for 30 sec, annealing at 58°C for 1 min and extension at 72°C for 2 min with a final extension at 72°C for 8 min. The PCR fragments were screened on a 1% agarose gel, purified with a QIAquick PCR purification kit (Qiagen), and both strands directly cycle-sequenced by the dideoxy-chain-termination method. Primers used for sequencing were the same as for PCR. Sequencing reactions were performed through the (French) national sequencing platform Genoscope, otherwise with an Applied Biosystems ABI PRISM BigDye terminator ready reaction cycle sequencing kit v3.0 following the manufacturer's instructions. Analyses were done on an ABI Prism 310 automated sequencer and edited sequences were deposited in the GeneBank database under the accession numbers EU250385-EU250471.

Sequences were edited in the SEQMAN II program, version 5.05 of the DNASTAR software package. In the case of the cyt b gene, they were aligned manually with published sequences of *Trematomus *whereas S7 sequences were aligned to each other using CLUSTALX software.

### Evaluation of intralocus recombination and the determination of gametic phases

an important assumption in the subsequent analyses is that all sites within studied loci share the same genealogical history, because recombination as a macromutation event may artificially introduce long branches into the genealogy [36]. To assess the prevalence of historical recombination within intron sequences we applied the "four-gamete test" [37]. Although this test evaluates only minimum number of intralocus recombination events, it is sensitive to the presence of recombination, which was our aim. We first used only S7 sequences from homozygous individuals to circumvent the potential misidentifications of gametic phases. Then, for any species dataset where the test suggested intralocus recombination, we discarded the sites left or right of the putative recombination events in order to retain the longest possible contiguous unrecombined sequence for subsequent analyses. We subsequently used the Bayesian statistical method implemented in the PHASE software [38] to reconstruct the gametic phases of heterozygous individuals. After treatment of intralocus recombination events, all the phase assessments had posterior probabilities greater than 95%.

### Phylogenetic analyses, molecular clocks and genetic diversity

For both loci, the minimum spanning networks among haplotypes within species-specific datasets were reconstructed by the Maximum Parsimony criterion using the algorithm of statistical parsimony [39] implemented in the TCS program, version 1.06, [40]. This approach evaluates the maximum number of nucleotide differences among haplotypes for which the assumptions of Maximum Parsimony are not violated, i.e. infers the probability that observed character changes defining the connections of haplotypes are due to no more than single mutations.

To compare the substitution rates between both loci and calibrate the molecular clocks, we tested for the rate constancy as the first step. Because both this analysis and molecular clock calibration should not be influenced by the recombination, we used the whole sequence of S7 intron. Using the hierarchical likelihood ratio test implemented in the MODELTEST program, version 3.06 [41], we chose the HKY 85 model of DNA substitution [42] with the gamma shape equal to 1.0126 and proportion of invariable sites = 0.4574 as suitable for the cytochrome b gene. For the S7 intron, the HKY 85 model with gamma shape parameter equal to 0.7804 was selected. We chose sequences of *Lycodes toyamensis *and *Eleginops maclovinus *as outgroups for the cyt b and S7 loci, respectively, and performed the relative rate tests implemented in HYPHY software [43] with the sequence substitution models described above. We then tested for the significance of differences in distances between the outgroup and every sampled haplotype on the one hand, and those between the outgroup and the haplotypes detected in a single *C. hamatus *specimen on the other.

Molecular clocks were calibrated by setting the divergence between Channichthyidae and Trematominae to 24 ± 0.5 Mya [[26]; most recent common ancestor of AFGP-bearing Antarctic notothenioids], and assessed the divergence rate by calculating the mean pairwise nucleotide divergence between haplotypes detected in the focal species and those from *C. hamatus *with the PAUP* software package, version 4.0b10 [44]. Because of rather low intraspecific variability in the cyt b gene, we used complete datasets demographic analyses (see below) and therefore estimated an average rate for the whole sequence for comparisons with the S7 locus. However, since most of the observed intraspecific variability was in third positions, which presumably mutate more rapidly, we also estimated their substitution rate to date the expansion events more accurately. To avoid the problem of mutation saturation, we first estimated the divergence rate at 1^st ^and 2^nd ^positions using *C. hamatus *as above and an appropriate model estimated by the MODELTEST. Subsequently, we estimated mean divergence within dataset composed of sequences from the four closely related focal species and compared it to estimated divergence at 3^rd ^positions to get an idea about relative mutation rate in both datasets.

The haplotype diversity (h) and mean nucleotide divergence (π) were estimated by ARLEQUIN software, v. 2.0 [45]. Contingency table and Wilcoxon-Mann-Whitney tests were used for significant differences among species reflecting different habitat preferences in both measures, respectively.

### Neutrality tests

since the inferences of historical demography may be seriously compromised by selective pressure on the study loci, we first performed the HKA test [46], which compares among loci the observed amounts of segregating polymorphisms within species to the number of differences between the species. It allows determining whether the amount of intraspecific polymorphism in any particular locus is significantly higher or lower than expected. In neutral case, the amount of polymorphisms and divergence are correlated in all loci. Since the test statistic is not expected to follow the χ^2 ^distribution in closely related species [47], we compared it to the distribution generated from 1000 coalescent simulations using the software HKA. We applied HKA tests to all four species using *T. scotii*, which is a basal species in the Trematominae radiation [48] as an outgroup. The McDonald-Kreitman test [49] was further used to assess whether the cyt b variability conforms to expectations under neutrality. Fisher's exact test implemented in the DnaSP software [50] was used to test whether the ratio of silent to replacement polymorphisms is the same as the ratio of silent to replacement fixed differences. Again, *T. scotii *was used as an outgroup.

### Inferences of past population size changes

Several approaches were employed to detect the traces of historical demographic changes in studied species:

The Tajima's test of neutrality [51] evaluates the significance of the amount of recent mutations. Significant excess of such mutations (negative Tajima's D values) may suggest selective sweep or population expansion events, while significant lack of recent mutations (positive values) could be attributed to balancing selection, population structure or decline.

Similarly, the method of Wakeley and Hey [52] implemented in SITES software [53] takes into account the distribution of polymorphisms in population and was used to fit the data to a model of sudden population expansion, which is characterised by parameters θ_ancestral_, θ_final _and τ (the time in generations scaled in mutation units since the size change took place). Using the substitution rate per locus per year (μ), the absolute time in years since the expansion (t) may be obtained as: t = τ/2μ.

From the tests considering the information from haplotype frequencies and number, we used Fu's F_s _test [54], which is expected to take negative values in expanded populations and is considered as one of the strongest in detecting traces of simulated logistic [54] as well as stepwise expansion models [17].

The mismatch distribution (MD) [55] takes into account the distribution of the number of pairwise mutation differences between sequences, which is expected to be irregular in shape in constant-size neutral model, while it is supposed to be unimodal in recently expanded populations. This test assumes a stepwise population growth model characterised by three parameters, i.e. θ_initial_, θ_final _and τ (the time elapsed since the size change took place; absolute time t = τ/2μ) [56]. The validity of this model was assessed by the parametric bootstrap approach using the sum of the squares of the deviations between observed and expected mismatch distributions as a statistic as implemented in ARLEQUIN. According to original method [56], the time of the putative population expansion (τ) was calculated from the mean and variance of the MD using the DNAsp. Since this method may lead to underestimates in case of rate heterogeneities, we also used the general non-linear least-squares approach [57] as implemented in ARLEQUIN, which also assesses the 95% confidence intervals around the estimated value of τ.

Low variability in mtDNA data of the benthic species resulted in frustratingly large C.I. of τ (see the Results section). We therefore applied the method of Saillard et al. [58] to estimate the time to the most recent common ancestor (MRCA) of *T. bernacchii *and *T. pennelli *sequences. This may be considered as an upper limit for the expansion date, since the onset of population expansion inferred from observed genealogies might hardly be older than genealogies themselves. We inferred the average distance of all individuals from the ancestral haplotype in the number of mutation steps (ρ = {n_1_l_1 _+...+ n_k_l_1_}/n, where n is the number of specimens and l is the length of the k^th ^branch expressed in mutation steps), and the variance of the estimate σ_H_^2 ^= {n_1_^2^l_1 _+...+ n_k_^2^l_1_}/n^2^. This estimates divided by per locus mutation rate give the absolute age of the genealogy and corresponding standard errors.

Markov Chain Monte Carlo (MCMC) approach taking into account phylogenetic relationships among haplotypes as implemented in FLUCTUATE v. 1.4 [18], was further used to assess the goodness of fit of the exponential growth model characterised by equation (θ_t _= θ_initial_^-gt^) to our data and to compute Maximum Likelihood (ML) based estimators of exponential growth rate (g). We set the transition/transversion ratio to 10 and starting value of θ was calculated as the Watterson [59] estimator of this parameter. When consistent estimates were obtained among runs with different number of steps in chains and initial values of g [60], we performed the final estimates running the program with five short chains of 15, 000 steps and two long chains of 400, 000 steps with a sampling increment of 20. Significances of g were assessed from the confidence intervals provided by FLUCTUATE [60]. Because of upward bias of g [18], we also adopted the conservative approach [61], where only values greater than three standard deviations of g are considered significant. We further combined both loci to perform the joined ML search for both parameters using LAMARC software, v. 2.0.2, since addition of unlinked loci is the most powerful way to reduce the bias of g. For the latter analysis, we assigned the effective population size for mtDNA as four times less than for the S7 intron and the mutation rate was set four times greater for the cyt b gene. We ran several analyses with varying random seeds and checked for consistency of results.

In addition to the above tests, we applied the approach of Galtier et al. implemented in the software SWEEP-BOTTLENECK [19] to detect significant traces of diversity reducing events, resulting from demographic (bottlenecks) or selective (sweeps) causes. Since the software assumes an equal effective population size for all loci, we couldn't run combined analysis to disentangle between both scenarios and only searched for significant deviations from a no-founder model (i.e. neutral model with a population of constant size) in individual loci. Since the test is applicable only to data where no more than two states are observed at any position and where both sequence and nucleotide sites give identical phylogenetic information (the suitability of the dataset is recognised by the software), we could not use it for *T. newnesi *and *P. borchgrevinki *mtDNA and *P. borchgrevinki *S7 datasets.

Geographical structure may affect the estimates of all the above tests, so we used Analysis of Molecular Variance (AMOVA) [62] implemented in ARLEQUIN to test for it. Since ARLEQUIN does not allow the correction of genetic distances according our best-fitting model of DNA substitution, we used the Kimura [63] method. We estimated the level of genetic structuration within populations, between populations within a region and between regions. We defined the regions according to traditional biogeographic separation of Antarctic Peninsula and continental Antarctica. Thus, all populations from the vicinity of the Ross Sea were grouped together and compared against samples taken from the vicinity of the Peninsula. We further measured the Φ_ST _[62] over all populations, which is analogous to Wright' F_ST_, but takes into account the divergence between haplotypes. We chose only *T. bernacchi *and *T. newnesi *for these analyses, since in the other two species we either managed to sample only two localities, or we obtained highly unbalanced sample numbers among populations and they both were concentrated within relatively small region. In nuclear datasets of all species we tested for significant departures from Hardy-Weinberg equilibria (HWE) using test implemented in the HWE package developed by J. Brusztowski, which uses the Markov Chain algorithm of Guo & Thompson [64] to correct for bias inherent in small sample sizes.

## Results

**Figure 2:**
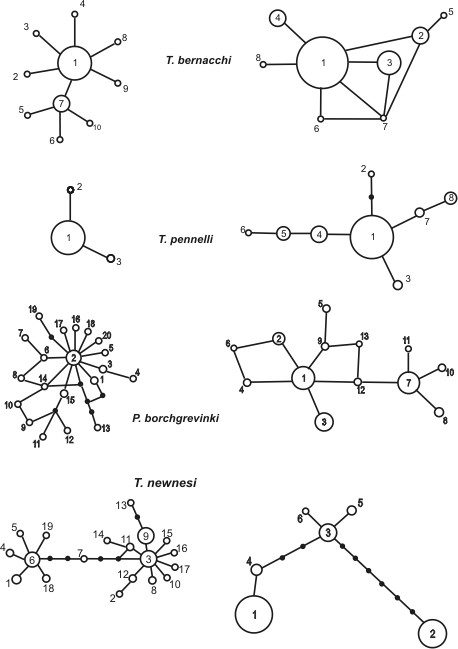
Unrooted networks constructed by statistical parsimony reconstructing the mutational relationships among sampled haplotypes (white circles) as well as putative intermediate steps (black circles). The circle sizes are proportional to observed frequencies of haplotypes. Left: networks based on cytochrome b sequences, Right : networks based on S7 intron sequences.

The summary of mtDNA and S7 data is given in the Table 2. As expected for a coding region, we observed no indels or stop codons in the cyt b dataset. We observed 1, 0, 1 and 2 recombination events in the *T. bernacchi, T. pennelli, P. borchgrevinki *and *T. newnesi *data respectively. We observed higher genetic diversity as well as nucleotide diversity in pelagic species relative to both benthic species for both markers. The differences between benthic and pelagic species in both measures were highly significant (P ~ 10^-16^). Concordantly, haplotype networks of the benthic species were rather simple consisting of one frequent haplotype surrounded by several rare haplotypes, compared to more complex cladograms and higher average distance among haplotypes in pelagic species (Figure 2).

**Table 2:** Diversity indices from sequences of cyt b and S7 intron.

*Cyt b*	L	N	PS	Hd (S.D.)	π (S.D.)
*Trematomus bernacchi*	468	10	9	0.482 (0.0719)	0.129% (0.115%)
*Trematomus pennelli*	500	3	2	0.1321 (0.0680)	0.025% (0.042%)
*Pagothenia borchgrevinki*	578	20	16	0.8770 (0.0532)	0.355% (0.228%)
*Trematomus newnesi*	483	13	15	0.9065 (0.0233)	0.687% (0.399%)
S7					
*Trematomus bernacchi*	307	8	7	0.5144 (0.0492)	0.201% (0.179%)
*Trematomus pennelli*	590	8	8	0.5180 (0.0609)	0.140% (0.112%)
*Pagothenia borchgrevinki*	456	13	9	0.8411 (0.0237)	0.3995% (0.258%)
*Trematomus newnesi*	299	7	9	0.7922 (0.0294)	0.741% (0.029%)

Number of haplotypes (N) was determined from alignments of sequences of length (L) from the individuals examined. The number of polymorphic sites (PS) is given for each species as well as the haplotype diversity (Hd) and the nucleotide diversity (π).

### Calibration of molecular clocks and relative mutation rates

Relative rate tests did not result in rejection of rate constancy in any comparison performed. The average pairwise divergence between *Chionodraco hamatus *and haplotypes sampled in the four Trematominae species was 0.232 (0.225 – 0.253) for the cyt b gene and 0.071 (0.061 – 0.08) for the S7 intron suggesting divergence rates of 0.98% and 0.28% per My in each marker respectively. The same divergence estimated from of the 1^st ^and 2^nd ^cyt b nucleotide positions was 0.059 (0.047 – 0.065) suggesting divergence rate of 0.246% per My. A comparison of pairwise divergences within the four-species datasets based on 1^st ^and 2^nd ^versus 3^rd ^positions suggested 14 times higher substitution rate at 3^rd ^positions equivalent to 3.44% per My.

### Neutrality tests

we detected no deviations from expectations under neutrality in the HKA test (*T. newnesi*, χ^2 ^= 1.33, P = 0.25; *T. bernacchi*, χ^2 ^= 0.0923, P = 0.76; *T. pennelli*, χ^2 ^= 2.11, P = 0.15; *P. borchgrevinki*, χ^2 ^= 0.0002, P = 0.98700). A McDonald-Kreitman test also revealed no signatures of selective pressure on the cyt b gene in any of the species (*T. bernacchi *P = 1.0, *T. pennelli *P = 1.0, *P. borchgrevinki *P = 0.445, *T. newnesi *P = 0.43).

### Population structure

We observed no significant deviations from Hardy-Weinberg equilibrium in either species (P-values were: *T. bernacchi *0.9, *T. pennelli *0.33, *P. borchgrevinki *0.31, *T. newnesi *0.99). At the nuclear locus, fixation indices (Φ_ST_) over all populations were insignificant (-0.0013 for *T. bernacchi *(P = 0.35) and 0.018 for *T. newnesi *(P = 0.15)) and AMOVA suggested that the within-population variance accounted for 100% and 98.2% of total molecular variance in each species respectively. At the cyt b locus, *T. newnesi *still showed no significant and negative Φ_ST _values over all populations (-0.021 (P = 0.8)) and AMOVA suggested that all the molecular variance is explained by within-population variances. The results for *T. bernacchii *were different however, suggesting that the South Shetlands and Ross Sea populations are divergent. We observed large and highly significant Φ_ST _over all populations (0.69916 (P << 0.05)). AMOVA suggested the differentiation between the South Shetland and Ross Sea populations accounted for 69.48% of total variance, while only 30.08% could be attributed to within-population variability, and less than 0.5% could be attributed to variation among populations within the Ross Sea region.

### Inferences of past population size changes

The results from the six tests are summarized in Table 3. Since we observed significant population structure in *T. bernacchii*, which may suggest separate refugia for populations from the South Shetlands and the vicinity of the Ross Sea (see below), we performed all analyses on a whole dataset of this species as well as separately on populations from the Ross Sea with a high overall sample size.

**Table 3:** Results of demographic analyses performed with various methods.

	*MD*	*W-H*	*Taj. D*	*Fu's F*	*FLUCTUATE*	*LAMARC*	*SWEEP-BOTT*
*Cyt b*							
*T. bernacchi*	θi = 0 (0–0.7), θ_*f *_= 550 (0.97–3890)	θ_*i *_= 0.21, θ_*f *_= 2.5	-1.8 *	-7.7 **	3969 (s.d. 199)‡	1008 (36–1974)	-11.9 vs.0 **
*T. bernacchi (Ross Sea)*	θ_i _= 0 (0–0.45), θ_f _= 0.356 (0–2365)	θ_i _= 0.0001, θ_f _= 19.	-2.18 **	-8.11**	10000 (s.d. 1670) ‡	4454 (3575–5625)	-10.57 vs. 0 **
*T. penelli*	θ_i _= 0.1 (0–0.34), θ_f _= 0.141 (0–776)	θ_i _= 0, θ_f _= 3.8	-1.31	-2.29 **	5717 (s.d. 745) ‡	2246 (16–4064)	-2.94 vs. 0.56 **
*P. borchgrevinki*	θ_*i *_= 0 (0–1.5), θ_*f *_= 2110 (15.8–7666)	θ_*i *_= 0, θ_*f *_= 15.1	-1.95 **	-19.04 **	8350 (s.d. 135) ‡	4407 (3158–4981)	*NA*
*T. newnesi*	θ_*i *_= 0.005 (0–2.1), θ_*f *_= 6.58 (3–1645)	θ_*i *_= 0, θ_*f *_= 5.2	-0.83	-7.81 *	889 (s.d. 116) ‡	482 (180–696)	*NA*
S7							
*T. bernacchi*	θ_i _= 0 (0–0.5), θ_f _= 552 (1.3–3678) **	θ_i _= 0, θ_f _= 2.6	-1.21	-3.95	1587 (s.d. 156)		-16.4 vs. 0 **
*T. bernacchi (Ross Sea)*	θ_i _= 0 (0–0.6), θ_f _= 492 (1–3406)**	θ_i _= 0, θ_f _= 2.3	-0.88	-2.16	1387 (s.d. 191) ‡		-10.4 vs. -7.3 *
*T. penelli*	θ_*i *_= 0.004 (0–0.7), θ_*f *_= 1.354 (0.1–4081)	θ_*i *_= 0, θ_*f *_= 1.0	-1.15	-2.88	1813 (s.d. 274) ‡		-22.02 vs. 0 **
*P. borchgrevinki*	θ_i _= 0 (0–0.8), θ_f _= 33.1 (5.6–6823)	θ_i _= 0, θ_f _= 2.38	-0.15	-2.77	1013 (s.d. 151) ‡		NA
*T. newnesi*	θ_i _= 0.006 (0–7.8), θ_f _= 4.08 (1.3–29)*	θ_i _= 11.6, θ_f _= 0	2.09*	3.97	33 (s.d. 86)		-26.1 vs. 24.2

For the Mismatch distribution (MD) and Wakeley and Hey's (W-H) methods, we indicate the pre- (θ_i_) and post- (θ_f_) expansion population parameters. Significant departures from expectation under the sudden expansion model of MD are indicated with asterisks (* means P < 0.05, ** < 0.01). Tajima's D and Fu's F_s _are indicated with their significance as above (in the latter case, * stands for P < 0.02 and ** for < 0.004). ML values of growth parameters estimated separately for each locus in FLUCTUATE are noted together with their standard deviation in parentheses. ‡ denotes cases where the zero growth value were excluded from the the 95% C.I. We further indicate the ML estimates of growth parameters for joined dataset as estimated by LAMARC together with 95% C.I. Finally, we also indicate the Log Likelihood values of no-founder and bottleneck models of Galtier et al. as estimated by SWEEP-BOTTLENECK software. Asterisks denote the significance of the LRT test as above. NA denotes cases, where this method was not applicable.

Except for nuclear data from *T. newnesi *all Tajima's D and Fu's F_s _coefficients were negative, which may be suggestive of population growth, although we observed significant deviation from the neutral model only in mtDNA. Similarly, Wakeley and Hey's [52] method indicated population growth in all datasets, except for *T. newnesi *S7, although the expected patterns under sudden population expansion were closely met only in the cyt b sequences from both benthic species. In remaining datasets we observed a poor fit with the expected values.

**Figure 3:**
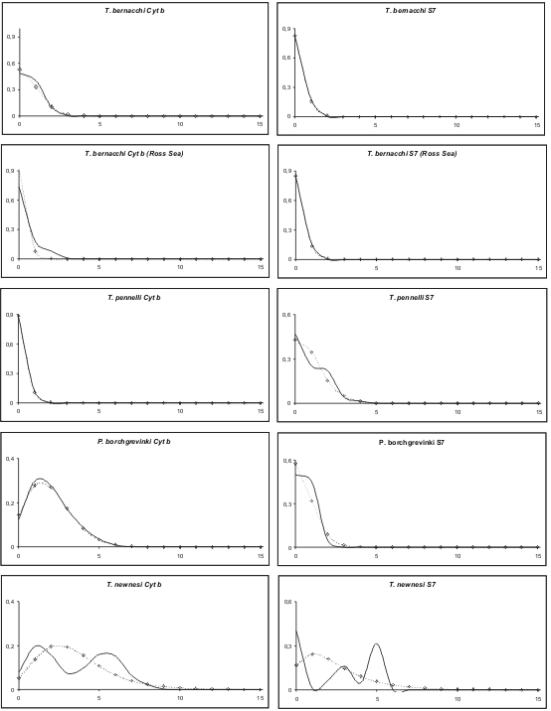
Mismatch distributions of observed (solid lines) pairwise among-individual comparisons as well as theoretical curves for sudden expansion model (dashed lines). For each species, the mismatch distribution of cyt b sequences is given on the left side, while the mismatch distribution of the S7 sequences is given at the right.

Mismatch distributions significantly deviated from expected distributions under the Rogers [56] model of sudden population expansion only in *T. bernacchii *and *T. newnesi *S7 sequences and are shown in (Figure 3). Both *T. newnesi *datasets had highest divergences of all species and a clearly multimodal curve, which is rather typical for populations of stable size [55].

The ML values of growth parameter g estimated separately for both loci using FLUCTUATE indicated significantly positive population growth in all cases except for *T. newnesi *S7 dataset, where the zero value couldn't be excluded from the estimated interval and standard deviation. Combined analyses of both loci resulted in significantly positive population growth in all species.

The Galtier et al. [19] test based on cyt b data suggested that both benthic species underwent a strong diversity reducing event, such as a bottleneck, occurring recently in *T. bernacchi *(in fact, the result suggested its occurrence 0 generations ago). Unfortunately, the low variability of *T. pennelli *cyt b prevented clear timing of the bottleneck, since after fixing the analysis for various values of the time parameter, we obtained very similar likelihood scores. This test further detected significant support for a recent (0.0001 × 2N_e_) strong bottleneck in both *T. bernacchi *and *T. pennelli *S7 datasets.

### Timing of the major demographic events

In addition to the above evidence of recent bottlenecks in benthic species, which nonetheless do not allow the comparison between both groups of species, the estimators of the parameter τ derived from MD [56] and the Wakeley and Hey's [52] method suggest much more recent expansion event in both benthic species than in the pelagic species at the cyt b (see Table 4). However, the C.I. of Schneider and Excoffier's [57] estimator of *T. bernacchi *expansion overlapped with that of *P. borchgrevinki *(although its mean value was about half that of the latter species). More importantly, when applied to *T. pennelli *and the Ross Sea population of *T. bernacchi*, Schneider and Excoffier estimator resulted in uninformative values with large C.I. In both cases, the Saillard et al [58] estimators of the times to MRCA stand in favour of more recent expansions of benthic species as the age of the *T. penelli *genealogy is estimated to 26.5 ± 1.6 kya and the *T. bernacchi *Ross Sea population to 61 ± 6 kya (the coalescence of the whole *T. bernacchi*'s genealogy was estimated to 166 ± 37 kya). These estimates changed to 22.5 ± 1.4 and 52 ± 5 when only 3^rd ^positions were considered.

**Table 4:** The ages of putative expansion events in mutation-time units (τ) and absolute time in kya (T) estimated by three methods, i.e. MD of [56] and [57] using software DNAsp and ARLEQUIN, respectively; and Wakeley and Hey's method in SITES (see the Materials and Methods section for explanation).

	MD (DNAsp)	MD (ARLEQUIN)	W-H
*Cyt b*			
*T. bernacchi*	τ = 0.631; T = 138	τ = 0.73 (0,22 – 1,1); T = 159 (47 – 236)	τ = 0.46; T = 100
*T. bernacchi (Ross Sea*	τ = 0.084; T = 18	τ = 3 (0.46 – 4.31); T = 654 (100 – 940)	τ = 0.44; T = 95
*T. penelli*	τ = 0.121; T = 25	τ = 3 (0.47 – 4.25); T = 612 (96 – 867)	τ = 0.123; T = 25
*P. borchgrevinki*	τ = 1.94; T = 350	τ = 1.94 (0.59 – 2.61); T = 350 (106 – 471)	τ = 1.97; T = 355
*T. newnesi*	τ = 2.15; T = 453	τ = 5.33 (1.65 – 9.97); T = 1125 (349 – 2111)	τ = 4.53; T = 956
Cyt b 3rd positions			
*T. bernacchi*	τ = 0.54; T = 100	τ = 0.66 (0.22 – 1); T = 122 (36 – 184)	τ = 0.654; T = 121
*T. bernacchi (Ross Sea)*	τ = 0.1; T = 19	τ = 2.9 (0.46 – 4.3); T = 537 (85 – 796)	τ = 0.29; T = 53
*T. penelli*	τ = 0.121; T = 21	τ = 3 (0.47 – 4.25); T = 520 (82–737)	τ = 0.12; T = 21
*P. borchgrevinki*	τ = 1.76; T = 267	τ = 1.78 (0.46 – 2.49); T = 270 (70 – 378)	τ = 1.8; T = 273
*T. newnesi*	τ = 1.38; T = 250	τ = 5.48 (1.5 – 11.8); T = 993 (272 – 2138)	τ = 3.96; T = 717
S7			
*T. bernacchi*	τ = 0.19; T = 218	τ = 0.72 (0.36 – 0.95); T = 839 (420 – 1107)	τ = 0.7; T = 815
*T. bernacchi (Ross Sea)*	τ = 0.16; T = 184	τ = 0.69 (0.37 – 0.96); T = 804 (441 – 1136)	τ = 0.66; T = 769
*T. penelli*	τ = 0.56; T = 338	τ = 1.515 (0.15 – 4.36); T = 917 (91 – 2640)	τ = 3.3; T = 2000
*P. borchgrevinki*	τ = 0.551; T = 419	τ = 2.02 (0.936 – 3); T = 1732 (802 – 2517)	τ = 2.7; T = 2116
*T. newnesi*	τ = 0.88; T = 1053	τ = 6.65 (2.27 – 15); T = 7957 (2716 – 17949)	τ = 0; T = 0

95% C.I. estimated by ARLEQUIN are indicated in parentheses.

The age of population expansions suggested by the nuclear marker were considerably older in all species. Although generally indicating more recent expansions in benthic species, disagreement among applied methods and ranges of C.I. (see Table 4), including the Saillard et al.'s estimates (data not shown), prevented any clear-cut conclusion. It should be stressed that for *T. bernacchi *and *T. newnesi *data, we could only use the estimates from SITES, which provides no confidence intervals, as their MD significantly differed from expectations of sudden expansion model assumed by this method.

## Discussion

### Comparison among markers and demographic interpretations

The extreme difficulties of sampling in Antarctica resulting in relatively low sample sizes and coverage, prevent any detailed conclusions about Trematominae population structure. Some aspects deserve discussion, however, especially due to the potential effect on demographic inferences. Whereas analysis of the S7 locus detected no deviations from panmixia in both species, analysis of mtDNA suggested a distinctive South Shetland population of *T. bernacchi*, which was previously postulated [65]. Such discrepancies between nuclear and cytoplasmic markers have been more or less frequently encountered in other studies, and may result from a number of mechanisms. Whereas some studies clearly stand in support of deterministic explanations, such as selective pressures or different evolution of sexes [66,67], other cases (including Antarctic toothfish [68]) were explained by a simpler stochastic effect of different coalescence dynamics of mtDNA and nuclear markers [e.g. [69-73]]. A four-fold smaller effective population size of mtDNA relative to nuclear loci makes it more sensitive to population oscillations [[73] and citations therein], and suggests that genetic drift will result in a faster population subdivision relative to a nuclear locus.

We prefer the latter explanation given the lack of evidence of sex-specific dispersal in Trematominae and no apparent signs of either direct or background selection on polymorphism in either locus. *Trematomus bernacchi*, in common with other benthic fish [74] shows a pronounced population structure on a continent-wide scale. Grounded ice sheets did not cover the whole shelf in the Ross Sea during LGM [75,76], which is therefore considered as a potential refugium for benthic biota in particular [77]. Together with a decrease of oceanic current activities during glacial periods [78] reducing passive larval dispersal [79,80], our data are concordant with fragmentation into separate refugia with subsequent independent population expansions. Our sampling is far from complete and the following interpretations are only very approximate. The observed patterns and assumption of geographic homogeneity inherent with the demographic inference tools, at least justify the analysis of the Ross Sea population as a separate entity. The South Shetlands population was not analysed separately due to the low sample size from this location.

Despite generally concordant signals of population expansion from both loci, demographic analyses indicated another potential conflict between mtDNA and nuclear markers: 1) We couldn't significantly reject the constant-size model in traditional methods of Tajima's D and Fu's F_s _in the S7 datasets despite negative values, 2) estimates of g were lower in nuclear genes and 3) inferred expansion dates were much more ancient in S7. Locus-specific selective sweeps may explain such discrepancies [[81] and citations therein], but our results make such an explanation unlikely. Whereas Tajima's D and Fu's F_s _are sensitive to demographic oscillations, we detected no signs of selection in the McDonald-Kreitman or HKA tests, which are not expected to be affected by population size changes. Furthermore, these traditional measures seem more conservative than ML methods, which also use phylogenetic information [19,61]. Our interpretation of concordant expansion signals is further strengthened by the similar results of Galtier et al. test for both datasets of benthic species. In addition, significantly positive population growth estimated independently from both loci was supported by the results of a combined analysis, which is known to effectively reduce the upward bias in estimates of g [18].

In our opinion, contrasting signals from cyt b and S7 markers with respect to expansion rates, times and the significance of MD, Tajima's D and Fu's F_s_, are better explained by the different properties of each marker and population histories that were not identical to the simple models assumed by demographic methods. Given that g is scaled by the locus-specific mutation rate [18], lower rates in S7 likely come from an approximately four-fold difference in mutation rate of both markers (M. Kuhner and L. Smith, pers. comm.). It also suggests that a faster-evolving locus like cyt b is likely to incorporate traces of more recent demographic events [82]. Furthermore, demographic histories of the studied species were probably more complex than simple exponential or stepwise expansion due to repeated climatic cycles during the Pleistocene. Rogers and Harpending [55] and Rogers [56] showed that in cases of complex population histories, the parameter τ correlates with the initial expansion, which would obscure the effects of later events (including bottlenecks) for some time. Nuclear markers with longer coalescent times relative to mtDNA are expected to accumulate the information of demographic history over longer periods and indicate older events. It is also possible that, compared to the cyt b, temporal changes in population growth rate attenuated "more ancient" signals of population expansion in S7 to a greater extent.

Clear discordance between cyt b and S7 was nonetheless observed in the *T. newnesi *data, where positive values of Tajima's D and Fu's F_s _(significant Tajima's D) may suggest population subdivision (albeit not detected in AMOVA, Φ_st _and HW analyses) or balancing selection in S7 (but see the discussion above). We also reanalysed *T. newnesi *data with exclusion of the South Shetland population to avoid the effect of any undetected population structure, but obtained almost identical results (data not shown). Alternatively, positive values may also indicate a drop in population size, which is further supported by the method of Wakeley and Hey and insignificance of g estimates. Although combined analysis of both loci suggested positive growth in this species, it was the lowest among all species and our data probably do not allow us to make strong conclusions about the demographic history in this case.

When looking at the time-scale of faster-evolving mtDNA, which also allows the comparison with other published studies, the expansion events in pelagic species seem more ancient compared to benthic ones (the expansion of *T. newnesi *– if any – has the oldest date) although confidence intervals were quite large and partly overlapping especially between the *P. borchgrevinki *and *T. bernacchi *complete dataset. Unresolved estimates of τ for *T. pennelli *and the *T. bernacchi *Ross Sea population based on Schneider and Excoffier's [57] method (compare with lower τ values estimated from whole *T. bernacchi *data where the MD was skewed more to the right) may suggest that there were too few informative sites in our data for this method to provide reliable estimates. In any case, we have shown that lower C.I. for expansion event in *P. borchgrevinki *is older than the age of whole genealogy of the Ross Sea population of *T. bernacchi *(the age of *T. pennelli *is even younger). Since all inferences of demographic histories were based in one or the other way on mutation spectre in observed data, it is not possible, in our opinion, to detect expansion times older than the data themselves, supporting a more recent expansion of benthic species.

In summary, while the demographic history of *T. newnesi *remains somewhat obscure, the data from remaining three species strongly indicate that they underwent significant population growth events during the Pleistocene. Given the results of Galtier et al. test, the proposed population expansions may be linked to recovery from recent strong bottlenecks at least in case both benthic feeders (this method does not indicate the beginning of the event however, as the same bottleneck effect may come from short and strong population reduction or a long but weak one). Given the time estimates from both loci, we assume that populations of these species were fluctuating during much of the Pleistocene. The more recent expansion times of benthic feeders assessed by means of mtDNA may suggest different reactions of each group to historical climatic events.

Using the comparative approach [61], there is no apparent distinction between benthic and pelagic species with respect to the expansion rate, since ML values of g were overlapping between both groups. Thus, we may not confirm the hypothesis that population oscillations were more dramatic in benthic species. Our data suggest that the benthic *T. bernacchi *stands at one extreme of the expansion rate estimates, whereas the pelagic *T. newnesi *stands at the other.

### Comparisons to other Southern Ocean organisms

Increasing numbers of studies of Northern Hemisphere marine fish suggest their populations have generally undergone important population size oscillations, which are interpreted in relation to Pleistocene changes of currents, sea-level or temperature [e.g. [83-89]]. Some species-specific ecological characteristics may nonetheless buffer the impact of climatic fluctuations on populations of marine organisms, while others, such as restriction to habitats prone to dessication or greater specialisation in diet and habitat preferences, make them more vulnerable to such changes [e.g. [90-93]].

In constrast, the scarcity of population genetic and phylogeographic studies on Southern Ocean marine organisms limits our understanding of Southern populations. Our finding of more recent expansion dates in benthic *Trematomus *suggest that habitat preference may have modified the impact of Pleistocene climatic shifts in agreement with the glacial-mediated habitat eradication hypothesis. However, absolute dating of demographic events and their relation to particular climatic events may be compromised by errors associated with molecular clock calibration and should be considered as approximate. On the other hand, such estimates may provide useful comparisons among species. Provided our calibration is correct, our data seem to provide a surprisingly good match of *T. pennelli *expansion to the onset of the last deglaciation, beginning about 25 kya but possibly as early as 30 kya in some parts of Antarctica [94]. The *T. bernacchi *expansion also probably happened during the last major glacial period (or during the last ~50 kya when considering the 3^rd ^cyt b position dataset). The best estimates from our data suggest that major population expansions of both pelagic Trematominae species by far predated the Holocene, and even the last interglacial, although, strictly speaking, the expansion of *P. borchgrevinki *might have occurred as recently as 70 kya according to 95% C.I..

A similar picture is suggested by data published so far, since other pelagic organisms, namely the krill *Euphausia superba *[22] and the silver fish *Pleuragramma antarcticum *[16], have most likely experienced populations expansions and/or bottlenecks by far predating the Holocene, dated to between 205 – 304 kya according to alternative molecular clocks and around 120 kya (48 – 142 kya according to 95% C.I.) in each species respectively. In their study of three benthic icefish (*Chionodraco*), Patarnello et al. [74] found high haplotype diversities, low divergences and negative values of Tajimas' D otherwise supportive for population bottlenecks. However, the insignificant values found in this study prevented the authors from reaching conclusions about the demographic history of these icefish. Reanalysing these data, we found in all three species smooth unimodal mismatch distributions and significantly negative values of Fu's F_s _in *C. hamatus*. In the remaining species, both Tajima's D and Fu's F_s _were significant at 10% level. We also found significantly positive values of g on the order of 10^2 ^in all three species (data available upon request). Using the same substitution rate as in [16], the population expansions in two of the three benthic species seem more recent compared to previously published data on pelagic feeders – most likely values suggested their onset approximately at 90, 47 and 25 kya in *C. hamatus, C. myersi *and *C. rastrospinosus*, respectively (95% C.I. suggested upper dates at 180, 95 and 90 kya, respectively).

To compare these data with our results we have to synchronise molecular clocks among the above studies [16,74] and this paper. This is because we calibrated the clocks by the separation of Channichthyidae and Nototheniidae dated to 24 Mya according to the fossil-calibrated molecular clock [26], while Zane et al. [16] used as calibration the split between *Eleginops maclovinus *and the rest of Notothenioidei dated approximately to the same time (22–25 Mya) because supposedly corresponding to the formation of the APF (surprisingly, that divergence was set to about 40 Mya using the fossil calibration [26]). On the one hand, the calibration in Zane et al. [16] refers to previous reports [5,95] using a calibration point of "perciform radiation" at 60 Mya, which is highly tentative, since we actually don't know what a perciform is [96]. This consequently led to a correspondence between the formation of the APF and the separation of *Eleginops *from the rest of the notothenioids. In our opinion, this event rather correlates with the diversification of AFGP-bearing notothenioids (i.e. the divergence time between *Pleuragramma *and *Chionodraco*), which corresponds to the data on Notothenioidei [26,97]. On the other hand, Near [26] based the calibration of his molecular clock on a single point with a fossil record and there have been doubts that this fossil really was a notothenioid. Both calibrations therefore suffer from some uncertainties.

Applying either one or the other calibration point to all fish datasets, we generally observed more recent expansion events in the benthic species relative to the pelagic organisms, except for *C. hamatus*. This indicates that habitat preferences affect the response to climatic shifts. Using the calibration date as in Zane et al. [16] (resulting in 1.6 to 1.8 times faster mutation rates compared to our calibration), the data would further suggest that population expansions of both benthic *Trematomus *correlate with the last glacial retreat.

On the other hand, the non-concordant signals in the rate and onset of population expansions within both groups of species complicate a clear-cut general conclusion. In addition to problems in using molecular clock estimates to compare the results for organisms as unrelated as fish and krill, it is possible that the sensitivity of species to glacial oscillations is determined by more complex traits than simple distinction between benthic and pelagic lifestyles. For example, some benthic species, like *T. bernacchi*, prefer shallow waters, feed mostly on benthic prey and use large sponges as spawning grounds, and thus require undisturbed habitats [33], while other benthic species such as *Chionodraco *spawn on rocks, consume large amounts of Euphausiids and pelagic fish, prefer greater depths and therefore might not necessarily be so vulnerable to glacial expansions.

## Conclusion

Our study represents the first multilocus attempt to link demographic events of several Antarctic marine species to their life history and climatic events. Although we observed discordant between-locus signals in relation to the time and rate of demographic events, our study suggest that most or all of the analysed species underwent population fluctuations during the Pleistocene. Based on mtDNA variability of the species studied in this work, it seems very likely that benthic species underwent a dramatic population bottleneck associated with the last glacial advance. If we trust our application of molecular clocks, it seems that at least *T. pennelli *started to recover as recently as in the Holocene. In contrast, pelagic populations either expanded much prior to LGM or remained stable in size. Taking into account previously published data, we still observed generally more recent population bottlenecks/expansions in benthic species relative to pelagic, but the onset of major demographic events seems asynchronous in both groups. We propose that the glacial-mediated habitat eradication hypothesis (see above) is a plausible explanation of genetic patterns observed in studied species, but requires further testing by comparing demographic histories of more species across taxonomic groups. This would also help to assess the ecological traits that best explain among-species differences. Such a study needs a better and more consistent calibration of molecular clocks. This is not an easy task for Southern Ocean biota given the chronic lack of fossils or other external calibration points.

## Abbreviations

AFGP, antifreeze glycoproteins; bp, base pairs; Cyt b Cytochrome b; HWE, Hardy-Weinberg equilibrium; LGM, last glacial maximum; MD, mismatch distribution; ML, maximum likelihood;

## Authors' contributions

KJ, GL, AV and CM designed the study and were involved in sampling. KJ, GL, CC and AC carried out the molecular work and the analyses. All authors contributed to the preparation of the manuscript. They read and approved the final version.

### Appendix

Absolute frequencies of haplotypes observed in studied localities

Cytochrome b (see Table 5).

**Table 5:** 

	CR	CA	TNB	CH	TA	SSh
*Trematomus bernacchi*						
Haplotype 1	8	9	10	5	10	1
Haplotype 2	0	0	1	0	0	0
Haplotype 3	0	0	0	0	1	0
Haplotype 4	0	1	0	0	0	0
Haplotype 5	1	0	0	0	0	0
Haplotype 6	1	0	0	0	0	0
Haplotype 7	0	0	0	0	0	10
Haplotype 8	0	1	0	0	0	0
Haplotype 9	1	0	0	0	0	0
Haplotype 10	0	0	0	0	0	1
*Trematomus pennelli*						
Haplotype 1	11	6	0	8	16	0
Haplotype 2	0	0	0	0	1	0
Haplotype 3	0	0	0	0	2	0
*Pagothenia borchgrevinki*						
Haplotype 1	0	2	0	0	0	0
Haplotype 2	0	10	0	0	2	0
Haplotype 3	0	2	0	0	0	0
Haplotype 4	0	0	0	0	1	0
Haplotype 5	0	1	0	0	0	0
Haplotype 6	0	1	0	0	0	0
Haplotype 7	0	1	0	0	0	0
Haplotype 8	0	1	0	0	0	0
Haplotype 9	0	1	0	0	0	0
Haplotype 10	0	1	0	0	0	0
Haplotype 11	0	1	0	0	0	0
Haplotype 12	0	1	0	0	0	0
Haplotype 13	0	1	0	0	0	0
Haplotype 14	0	1	0	0	0	0
Haplotype 15	0	1	0	0	1	0
Haplotype 16	0	1	0	0	0	0
Haplotype 17	0	1	0	0	0	0
Haplotype 18	0	0	0	0	1	0
Haplotype 19	0	1	0	0	0	0
Haplotype 20	0	1	0	0	0	0
*Trematomus newnesi*						
Haplotype 1	0	0	0	0	3	0
Haplotype 2	0	0	0	0	1	0
Haplotype 3	0	1	0	1	4	4
Haplotype 4	0	1	0	0	1	0
Haplotype 5	0	0	0	0	1	0
Haplotype 6	0	2	0	3	1	1
Haplotype 7	0	0	0	0	1	0
Haplotype 8	0	0	0	0	1	0
Haplotype 9	0	3	0	3	3	0
Haplotype 10	0	0	0	0	1	1
Haplotype 11	0	1	0	0	0	1
Haplotype 12	0	1	0	0	0	1
Haplotype 13	0	0	0	1	0	0
Haplotype 14	0	0	0	0	0	1
Haplotype 15	0	0	0	0	0	1
Haplotype 16	0	0	0	0	0	1
Haplotype 17	0	0	0	0	0	1
Haplotype 18	0	0	0	0	0	2
Haplotype 19	0	0	0	0	0	1
S7:						
	CR	CA	TNB	CH	TA	SSh
*Trematomus bernacchi*						
Haplotype 1	13	16	13	8	19	15
Haplotype 2	2	1	5	0	0	2
Haplotype 3	4	3	3	1	2	2
Haplotype 4	3	1	1	1	0	3
Haplotype 5	0	0	0	0	0	1
Haplotype 6	0	0	0	0	0	1
Haplotype 7	0	1	0	0	0	0
Haplotype 8	0	0	0	0	1	0
*Trematomus pennelli*						
Haplotype 1	17	8	0	11	24	0
Haplotype 2	0	0	0	0	1	0
Haplotype 3	0	1	0	0	2	0
Haplotype 4	3	2	0	2	3	0
Haplotype 5	1	0	0	2	4	0
Haplotype 6	0	0	0	1	0	0
Haplotype 7	0	0	0	0	3	0
Haplotype 8	1	1	0	0	1	0
*Pagothenia borchgrevinki*						
Haplotype 1	0	15	0	0	3	0
Haplotype 2	0	5	0	0	1	0
Haplotype 3	0	11	0	0	1	0
Haplotype 4	0	2	0	0	0	0
Haplotype 5	0	2	0	0	0	0
Haplotype 6	0	1	0	0	0	0
Haplotype 7	0	12	0	0	4	0
Haplotype 8	0	2	0	0	1	0
Haplotype 9	0	2	0	0	0	0
Haplotype 10	0	2	0	0	0	0
Haplotype 11	0	1	0	0	0	0
Haplotype 12	0	2	0	0	0	0
Haplotype 13	0	1	0	0	0	0
*Trematomus newnesi*						
Haplotype 1	0	10	0	6	20	16
Haplotype 2	0	8	0	5	5	8
Haplotype 3	0	0	0	5	7	0
Haplotype 4	0	0	0	0	1	3
Haplotype 5	0	0	0	0	1	0
Haplotype 6	0	0	0	0	0	3

## References

[B1] Kock KH (1992). Antarctic Fish and Fisheries.

[B2] Clarke A, Barnes DKA, Hodgson DA (2005). How isolated is Antarctica?. Trends Ecol Evol.

[B3] Antezana T (1999). Plankton of southern Chilean fjords: trends and linkages. Sci Mar.

[B4] Hodgson DA, Vyverman W, Tyler PA (1997). Diatoms of meromictic lakes adjacent to the Gordon River, and of the Gordon River estuary in south-west Tasmania. Biblio Diatomol.

[B5] Bargelloni L, Marcato S, Zane L, Patarnello T (2000). Mitochondrial phylogeny of notothenioids: a molecular approach to Antarctic fish evolution and biogeography. Syst Biol.

[B6] Patarnello T, Bargelloni L, Varotto V, Battaglia B (1996). Krill evolution and the Antarctic Ocean currents: evidence of vicariant speciation as inferred by molecular data. Mar Biol.

[B7] Held C (2000). Phylogeny and Biogeography of Serolid Isopods (Crustacea, Isopoda, Serolidae) and the Use of Ribosomal Expansion Segments in Molecular Systematics. Mol Phyl Evol.

[B8] Page TJ, Linse K (2002). More evidence of speciation and dispersal across the Antarctic Polar Front through molecular systematics of Southern Ocean Limatula (Bivalvia: Limidae). Polar Biol.

[B9] Lorza AN, Held C (2004). A preliminary molecular and morphological phylogeny of the Antarctic Epimeriidae and Iphimediidae (Crustacea, Amphipoda). Mol Phyl Evol.

[B10] Hambrey MJ, Barrett P, Kennett JP, Warnke DA (1993). Cenozoic sedimentary and climaticrecord, Ross Sea region, Antarctica. The Antarctic paleoenvironment: a perspective on globalchange Part two.

[B11] Thatje S, Hillenbrand C-D, Larter R (2005). On the origin of Antarctic marine benthic community structure. Trends Ecol Evol.

[B12] Eastman JT, Clarke A, di Prisco G, Pisano E, Clarke A (1998). Radiations of Antarctic and non-Antarctic fish. Fishes of Antarctica: A Biological Overview.

[B13] Anderson RF, Chase Z, Fleisher MQ, Sachs J (2002). The Southern Ocean's biological pump during the Last Glacial Maximum. Deep-Sea Research Part II Topical Studies in Oceanography.

[B14] Charles CD, Froelich PN, Zibello MA, Mortlock RA, Morley JJ (1991). Biogenic opal in Southern Ocean sediments over the last 450 000 years: implications for surface water chemistry and circulation. Paleoceanography.

[B15] Hayward TL (1997). Pacific ocean climate change: atmospheric forcing, ocean circulation and ecosystem response. Trends Ecol Evol.

[B16] Zane L, Marcato S, Bargelloni L, Bortolotto E, Papetti C, Simonato M, Varotto V, Patarnello T (2006). Demographic history and population structure of the Antarctic silverfish. Pleuragramma antarcticum Mol Ecol.

[B17] Ramos-Onsins SE, Rozas J (2002). Statistical Properties of New Neutrality Tests Against Population Growth. Mol Biol Evol.

[B18] Kuhner MK, Yamato J, Felsenstein J (1998). Maximum likelihood estimation of population growth rates based on the coalescent. Genetics.

[B19] Galtier N, Depaulis F, Barton NH (2000). Detecting bottlenecks and selective sweeps from DNA sequence polymorphism. Genetics.

[B20] Stevens MI, Hogg ID (2003). Long-term isolation and recent range expansion from glacial refugia revealed for the endemic springtail *Gomphiocephalus hodgsoni *from Victoria Land, Antarctica. Mol Ecol.

[B21] Roeder AD, Marshall RK, Mitchelson AJ, Visagathilagar T, Ritchie PA, Love DR, Pakai TJ, Partlan HCMC, Murray ND, Robinson NA, Kerry KR, Lambert DM (2001). Gene flow on the ice: genetic differentiation among Adélie penguin colonies around Antarctica. Mol Ecol.

[B22] Zane L, Ostellari L, Maccatrozzo L, Bargelloni L, Battaglia B, Patarnello T (1998). Molecular evidence for genetic subdivision of Antarctic krill (Euphausia superba Dana) populations. Proc R Soc Lond B.

[B23] Eastman JT, McCune AR (2000). Fishes on the Antarctic continental shelf: evolution of a marine species flock?. Journal of Fish Biology.

[B24] Chen L, DeVries AL, Cheng C-HC (1997). Convergent evolution of antifreeze glycoproteins in Antarctic notothenioid fish and Arctic cod. Proceedings of the National Academy of Sciences of the USA.

[B25] Bargelloni L, Ritchie PA, Battaglia B, Lambert DM, Meyer A (1994). Molecular Evolution at Subzero Temperatures: Mitochondrial and Nuclear Phylogenies of Fishes from Antarctica (Suborder Notothenioidei), and the Evolution of Antifreeze Glycopeptides. Mol Biol Evol.

[B26] Near JT (2004). Estimating divergence times of notothenioid fishes using a fossilcalibrated molecular clock. Antarctic Science.

[B27] Chen W-J, Bonillo C, Lecointre G, di Prisco G, Pisano E, Clarke A (1998). Phylogeny of the Channichthyidae (Notothenioidei, Teleostei) based on two mitochondrial genes. Fishes of Antarctica: A Biological Overview.

[B28] Derome N, Chen WJ, Dettai A, Bonillo C, Lecointre G (2002). Phylogeny of Antarctic dragonfishes (Bathydraconidae, Notothenioidei, Teleostei) and related families based on their anatomy and two mitochondrial genes. Mol Phylogenet Evol.

[B29] Ritchie PA, Bargelloni L, Meyer A, Taylor JA, Macdonald JA, Lambert DM (1996). Mitochondrial phylogeny of trematomid fishes (Nototheniidae, Perciformes) and the evolution of Antarctic fish. Mol Phylogenet Evol.

[B30] Near TJ, Pesavento JJ, Cheng CC (2003). Mitochondrial DNA, morphology, and the phylogenetic relationships of Antarctic ice fishes (Notothenioidei:Channichthyidae). Mol Phylogenet Evol.

[B31] Bargelloni L, Lecointre G, di Prisco G, Pisano E, Clarke A (1998). Four years in notothenioid systematics: A molecular perspective. Fishes of Antarctica: A Biological Overview.

[B32] Eastman JT (1993). Antarctic Fish Biology: Evolution in a Unique Environment.

[B33] DeWitt HH, Heemstra PC, Gon O, Gon O, Heemstra PC (1990). Nototheniidae. Fishes of the Southern Ocean.

[B34] Ekau W, di Prisco G, Pisano E, Clarke A (1991). Morphological adaptations and mode of life in High Antarctic fish. Fishes of Antarctica, A biological Overview.

[B35] Chov S, Hazama K (1998). Universal PCR primers for S7 ribosomal protein gene introns in fish. Mol Ecol.

[B36] Wall JD (2003). Estimating ancestral population sizes and divergence times. Genetics.

[B37] Hudson RR, Kaplan NL (1985). Statistical properties of the number of recombination events in the history of a sample of DNA sequences. Genetics.

[B38] Stephens M, Smith NJ, Donnelly P (2001). A new statistical method for haplotype reconstruction from population data. American Journal of Human Genetics.

[B39] Templeton AR, Crandall KA, Sing CF (1992). A cladistic analysis of phenotypic association with haplotypes inferred from restriction endonuclease mapping and DNA sequence data. III. Cladogram estimation. Genetics.

[B40] Clement M, Posada D, Crandall KA (2000). TCS: a computer program to estimate gene genealogies. Mol Ecol.

[B41] Posada D, Crandall KA (1998). MODELTEST: testing the model of DNA substitution. Bioinformatics.

[B42] Hasegawa M, Kishino H, Yano T (1985). Dating of the human-ape splitting by molecular clock of mitochondrial DNA. J Mol Evol.

[B43] Kosakovsky Pond SL, Frost SDW, Muse SV (2005). HyPhy: hypothesis testing using phylogenies. Bioinformatics.

[B44] Swofford DL (1999). PAUP*. Phylogenetic Analysis Using Parsimony (*and other methods). Version 4.0.

[B45] Schneider S, Roessli D, Excoffier L (2000). Arlequin: A software for population genetics data analysis. Ver 2.0.

[B46] Hudson RR, Kreitman M, Aguadé M (1987). A test of neutral molecular evolution based on nucleotide data. Genetics.

[B47] Hilton H, Kliman RM, Hey J (1994). Using hitchhiking genes to study adaptation and divergence during speciation within the *Drosophila melanogaster *complex. Evolution.

[B48] Sanchez S, Dettaï A, Bonillo C, Ozouf-Costaz C, Detrich B, Lecointre G (2006). Molecular and morphological Phylogenies of the Nototheniidae, with on taxonomic focus on the Trematominae. Polar Biology.

[B49] McDonald JH, Kreitman M (1991). Adaptive protein evolu tion at the Adh Locus in Drosophila. Nature.

[B50] Rozas J, Rozas R (1999). DnaSP version 3: an integrated program for molecular population genetics and molecular evolution analysis. Bioinformatics.

[B51] Tajima F (1989). Statistical Method for Testing the Neutral Mutation Hypothesis by DNA Polymorphism. Genetics.

[B52] Wakeley J, Hey J (1997). Estimating ancestral population parameters. Genetics.

[B53] Hey J, Wakeley J (1997). A coalescent estimator of the population recombination rate. Genetics.

[B54] Fu YX (1997). Statistical Tests of Neutrality of Mutations Against Population Growth, Hitchhiking and Background Selection. Genetics.

[B55] Rogers AR, Harpending H (1992). Population growth makes waves in the distribution of pairwise genetic differences. Mol Biol Evol.

[B56] Rogers A (1995). Genetic evidence for a Pleistocene population explosion. Evolution.

[B57] Schneider S, Excoffier L (1999). Estimation of demographic parameters from the distribution of pairwise differences when the mutation rates vary among sites:application to human mitochondrial DNA. Genetics.

[B58] Saillard J, Forster P, Lynnerup N, Bandelt HJ, Norby S (2000). mtDNA variation among Greenland Eskimos: The edge of the Beringian expansion. American Journal of Human Genetics.

[B59] Watterson GA (1975). On the number of segregating sites in genetical models without recombination. Theor Popul Biol.

[B60] Kuhner MK, Salemi M, Vandamme A-M (2003). LAMARC: estimating population genetic parameter from molecular data. The Phylogenetic Handbook; a practical approach to DNA and protein phylogeny.

[B61] Lessa EP, Cook JA, Patton JL (2003). Genetic footprints of demographic expansion in North America, but not Amazonia, during the Late Quaternary. Proceedings of the National Academy of Sciences, USA.

[B62] Excoffier L, Smouse P, Quattro J (1992). Analysis of mole cular variance inferred from metric distances among DNA haplotypes: application to human mitochondrial DNA restriction data. Genetics.

[B63] Kimura M (1980). A simple method for estimating evolutionary rates of base substitutions through comparative studies of nucleotide sequences. J Mol Evol.

[B64] Guo SW, Thompson EA (1992). Performing the Exact Test of Hardy-Weinberg Proportion for Multiple Alleles. Biometrics.

[B65] Bernardi G, Goswami U (1997). Molecular evidence for cryptic species among the Antarctic fish Trematomus bernacchii and Trematomus hansoni. Antarctic Science.

[B66] Castella V, Ruedi M, Excoffier L (2001). Contrasted patterns of mitochondrial and nuclear structure among nursery colonies of the bat *Myotis myotis*. J Evol Biol.

[B67] Wen B, Li H, Lu D, Song X, Zhang F, He Y, Li F, Gao Y, Mao X, Zhang L, Qian J, Tan J, Jin J, Huang W, Deka R, Su B, Chakraborty R, Jin L (2004). Genetic evidence supports demic diffusion of Han culture. Nature.

[B68] Shaw GP, Arkhipin AI, Al-Khairula H (2004). Genetic structuring of Patagonian toothfish populations inthe Southwest Atlantic Ocean: the effect of the Antarctic Polar Front and deep-water troughs as barriers to genetic exchange. Molecular Ecology.

[B69] Seddon JM, Santucci F, Reeve NJ, Hewitt GM (2001). DNA footprints of European hedgehogs,*Erinaceus europaeus *and *E. concolor*: Pleistocene refugia, postglacial expansion and colonization routes. Molecular Ecology.

[B70] Berggren KT, Ellegren H, Hewitt GM, Seddon JM (2005). Understanding the phylogeographic patterns of European hedgehogs,*Erinaceus concolor *and *E. europaeus *using the MHC. Heredity.

[B71] Monsen KJ, Blouin MS (2003). Genetic structure in a montane ranid frog: restricted gene flow and nuclear-mitochondrial discordance. Molecular Ecology.

[B72] Ludwig A, Congiu L, Pitra C, Fickel J, Gessner J, Fontana F, Patarnello T, Zane L (2003). Nonconcordant evolutionary history of maternal and paternal lineages in Adriatic sturgeon. Molecular Ecology.

[B73] Bensch S, Irwin DE, Irwin JH, Kvist L, Akesson S (2006). Conflicting patterns of mitochondrial and nuclear DNA diversity in *Phylloscopus *warblers. Molecular Ecology.

[B74] Patarnello T, Marcato S, Zane L, varotto V, Bargelloni L (2003). Phylogeography of the Chionodraco genus (Perciformes, Channichthydae)in the Southern Ocean. Mol Phylogenet Evol.

[B75] Licht KJ, Jennings AE, Andrews JT, Williams KM (1996). Chronology of late Wisconsin ice retreat from the western Ross Sea, Antarctica. Geology.

[B76] Domacke W, Jacobson EA, Shipp S, Anderson JB (1999). Late Pleistocene-Holocene retreat of the West Antarctic ice-sheet system in the Ross Sea: Part 1 – geophysical results. Geol Soc Am Bull.

[B77] Dayton PK, Oliver JS (1977). Antarctic soft-bottom benthos in oligotrophic and eutrophic environments. Science.

[B78] Kojima S, Segawa R, Hayashi I (1997). Genetic   differentiation among populations of the Japanese turban shell Turbo (Batillus) cornutus corresponding to warm currents. Ma Ecol Prog Ser.

[B79] Stepien CA (1999). Phylogeographical Structure of the Dover Sole Microstomus Pacificus: the Larval Retention Hypothesis and Genetic Divergence Along the Deep Continental Slope of the Northeastern Pacific Ocean. Mol Ecol.

[B80] Hohenlohe PA (2004). Limits to gene flow in marine animals with planktonic larvae: models of *Littorina *species around Point Con-ception, California. Biol J Linn Soc.

[B81] Ometto L, Glinka S, De Lorenzo D, Stephan W (2005). Inferring the Effects of Demography and Selection on *Drosophila melanogaster *Populations from a Chromosome-Wide Scan of DNA Variation. Mol Biol Evol.

[B82] Harpending HC, Batzer MA, Gurven M, Jorde JB, Rogers AR, Sherry ST (1998). Genetic traces of ancient demography. Proc Natl Acad Sci USA.

[B83] Grant WS, Bowen BW (1998). Shallow population histories in deep evolutionary lineages of marine fishes: insights from sardines and anchovies and lessons for conservation. Journal of Heredity.

[B84] Avise JC (2000). Phylogeography.

[B85] Lecomte FL, Grant WS, Dodson JJ, Rodriguez-Sanchez R, Bowen BW (2004). Living with uncertainty: genetic imprints of climate shifts in East Pacific anchovy(*Engraulis mordax*) and sardine (*Sardinops sagax*). Mol Evol.

[B86] Zardoya R, Castilho R, Grande C (2004). Differential population structuring of two closely related fish species, the mackerel *(Scomber scombrus) *and the chub mackerel *(Scomber japonicus)*, in the Mediterranean Sea. Mol Ecol.

[B87] Vinas J, Bremer JA, Pla C (2004). Phylogeography of the Atlantic bonito *(Sarda sarda) *in the northern Mediterranean:the combined effects of historical vicariance, population expansion, secondary invasion, and isolation by distance. Mol Phylogenet Evol.

[B88] Carlsson J, Jan RMC, Pindaro D-J, Carlsson JLM, Boles SB, Gold JR, Graves JE (2004). Microsatellite and mitochondrial DNA analyses of Atlantic bluefin tuna (*Thunnus thynnus thynnus*) population structure in the Mediterranean Sea. Mol Ecol.

[B89] Ely B, Viñas J, Bremer JRA, Black D, Lucas L, Covello K, Labrie AV, Thelen E (2004). Consequences of the historical demography on the global population structure of two highly migratory cosmopolitan marine fishes: the yellowfin tuna *(Thunnus albacare *s) and the skipjack tuna *(Katsuwonus pelami*s). BMC Evolutionary Biology.

[B90] Wares JP, Cunningham CW (2001). Phylogeography and historical ecology of the North Atlantic intertidal. Evolution.

[B91] Kaustuv R, Jablonski D, Valentine JW (2001). Climate change, species range limits and body size in marine bivalves. Ecology Letters.

[B92] Fauvelot C, Bernardi G, Planes S (2003). Reductions in the mitochondrial diversity of corale reef fish provide evidence of population bottlenecks resulting from Holocene sea-level change. Evolution.

[B93] Hickerson MJ, Cunningham CW (2005). Contrasting quaternary histories in ecologically divergent sister pair of low-dispersing intertidal fish (*Xiphister*) revealed by multilocus DNA analyses. Evolution.

[B94] Anderson JB, Shipp SS, Lowe AL, Smith Wellner J, Mosola AB (2002). The Antarctic ice sheet during the last glacial maximum and its subsequent retreat history: a review. Quat Sci Rev.

[B95] Bargelloni L, Zane L, Derome N, Lecointre G, Patarlello T (2000). Molecular zoogeography of Antarctic euphausiids and notothenioids: from species phylogenies to intraspecific patterns of genetic variation. Antarctic Science.

[B96] Nelson JS (2006). Fishes of the World.

[B97] Near T, Pesavento J, Cheng CH (2004). Phylogenetic investigations of Antarctic notothenioid fishes (Perciformes: Notothenioidei) using complete gene sequences of the mitochondrial encoded 16SrRNA. Mol Phylogenet Evol.

